# Neuropsychological effects of occupational exposure to organic solvents: A study of 37 cases

**DOI:** 10.1192/j.eurpsy.2023.2043

**Published:** 2023-07-19

**Authors:** A. Moussa, D. Brahim, N. Mechergui, H. Ziedi, W. Ayed, S. Ernez, I. Youssef, N. Ladhari

**Affiliations:** 1 Occupational pathology and fitness for work, Charles Nicolle Hospital, Tunis, Tunisia

## Abstract

**Introduction:**

Occupational exposure to organic solvents can have multiple health effects for exposed employees.Neuropsychic effects represent an important part of these effects and have a significant impact on patients’ ability to work

**Objectives:**

- To describe the socio-professional and medical characteristics of workers exposed to organic solvents
- To screen among the study population for neuropsychological effects related to an organic psychosyndrome using the Q16 questionnaire.

**Methods:**

A retrospective descriptive study of workers exposed to organic solvents, who were referred to the occupational medicine department of Charles Nicolle Hospital in Tunis for a medical assessment of their fitness for work over the period from 2016 to 2022. The socio-professional data were collected from the medical records. The Swedish Q16 questionnaire in its French version was used to screen for neuropsychological signs of organic psycho-syndrome.

**Results:**

A total of 37 workers were included. The mean age was 45.38 ± 8.63 years with a clear male predominance (77%). The mean occupational seniority was 21.39 ± 11.11 years. The average duration of the occupational exposure to organic solvents was 18.25 ± 11.29 years. The most represented sectors of activity were the plastics industry (11%), the automotive industry (19%), the carpentry sector (14%) and the aeronautics sector (9%). Our population was represented by polyvalent workers in 49% of cases and by painter in 24% of cases. Psychiatric history was noted in only one case. The main functional signs reported by the workers were wheezing dyspnea with breathing difficulties (13%) and headaches (11%).

The Q16 questionnaire was found to be positive in 65% of the cases, with a higher rate of positivity for the items relating to unusual fatigue (73%), irritability for no particular reason (67%), short memory (64%) and headaches (58%). Acquired dyschromatopsia detected by a Lanthony test was found in 39% of the cases, 23% of which was associated with a positive Q16 questionnaire. Additional exploration by specific psychotechnical tests was carried out in five cases, all of which came back positive with significant attentional and cognitive impairment.

A declaration of an occupational disease according to the Table n°23 (Halogenated derivatives of aliphatic hydrocarbons) and Table n°40 (other liquid organic solvents for professional use) of the Tunisian list of occupational diseases eligible for compensation was made in three and two cases respectively. A definitive exemption from exposure to organic solvents was indicated for all workers with a positive Q16 questionnaire.

**Conclusions:**

Exposure to organic solvents is a risk encountered in various occupational sectors. Thus, education of the employees to the dangers encountered with a reinforcement of the collective and individual technical protection means are essential in order to avoid their detrimental effects on health.

**Disclosure of Interest:**

None Declared

